# 5-(2-Fur­yl)-3-methyl-1-(3-nitro­phen­yl)-4,5-dihydro-1*H*-pyrazole

**DOI:** 10.1107/S1600536809031419

**Published:** 2009-08-15

**Authors:** Jun-qiang Chen, He-ping Li, Chang-shan Huang, Jin-ying Wu

**Affiliations:** a Energy Research Institute Co Ltd, Henan Academy of Sciences, Zhengzhou 450000, People’s Republic of China; b School of Chemistry and Biological Engineering, Guilin University of Technology, People’s Republic of China

## Abstract

In the title compound, C_14_H_13_N_3_O_3_, the pyrazoline ring assumes an envelope conformation with the furanyl-bearing C atom at the flap position. The dihedral angle between the furan and nitrobenzene rings is 84.40 (9)°. Weak inter­molecular C—H⋯O hydrogen bonding is present in the crystal structure.

## Related literature

For applications of pyrazoline derivatives, see: Hatheway *et al.* (1978 ▶); Mahajan *et al.* (1991 ▶); Sobczak & Pawlaczyk (1998 ▶).
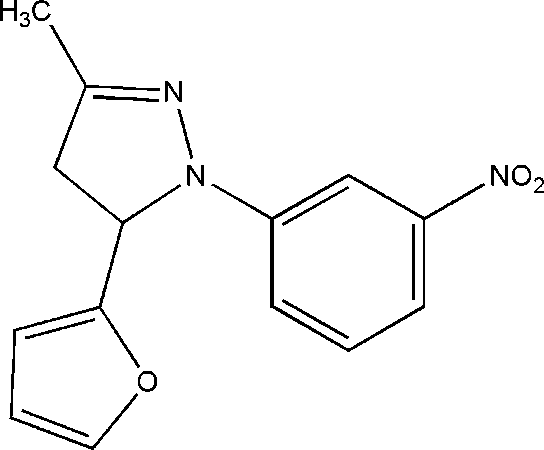


## Experimental

### 

#### Crystal data

C_14_H_13_N_3_O_3_
*M* *_r_* = 271.27
Triclinic, 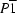

*a* = 6.2089 (2) Å
*b* = 7.8581 (3) Å
*c* = 14.3800 (4) Å
α = 105.764 (2)°
β = 97.054 (2)°
γ = 96.944 (2)°
*V* = 661.31 (4) Å^3^
*Z* = 2
Mo *K*α radiation
μ = 0.10 mm^−1^
*T* = 296 K
0.31 × 0.15 × 0.10 mm

#### Data collection

Bruker SMART CCD area-detector diffractometer
Absorption correction: none
9707 measured reflections
2590 independent reflections
1778 reflections with *I* > 2σ(*I*)
*R*_int_ = 0.036

#### Refinement

*R*[*F* ^2^ > 2σ(*F* ^2^)] = 0.044
*wR*(*F* ^2^) = 0.121
*S* = 1.10
2590 reflections
181 parameters
H-atom parameters constrained
Δρ_max_ = 0.14 e Å^−3^
Δρ_min_ = −0.21 e Å^−3^

### 

Data collection: *SMART* (Bruker, 1998 ▶); cell refinement: *SAINT* (Bruker, 1998 ▶); data reduction: *SAINT*; program(s) used to solve structure: *SHELXS97* (Sheldrick, 2008 ▶); program(s) used to refine structure: *SHELXL97* (Sheldrick, 2008 ▶); molecular graphics: *ORTEP-3 for Windows* (Farrugia, 1997 ▶); software used to prepare material for publication: *WinGX* (Farrugia, 1999 ▶).

## Supplementary Material

Crystal structure: contains datablocks global, I. DOI: 10.1107/S1600536809031419/xu2580sup1.cif

Structure factors: contains datablocks I. DOI: 10.1107/S1600536809031419/xu2580Isup2.hkl

Additional supplementary materials:  crystallographic information; 3D view; checkCIF report

## Figures and Tables

**Table 1:** Hydrogen-bond geometry (Å, °)

*D*—H⋯*A*	*D*—H	H⋯*A*	*D*⋯*A*	*D*—H⋯*A*
C12—H12*A*⋯O1^i^	0.93	2.51	3.311 (2)	144

Symmetry code: (i) 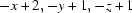.

**Fig. 1.**
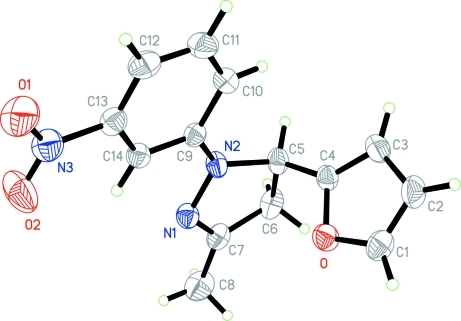
The molecular structure of the title compound. Displacement ellipsoids are drawn at the 30% probability level.

**Fig. 2.**
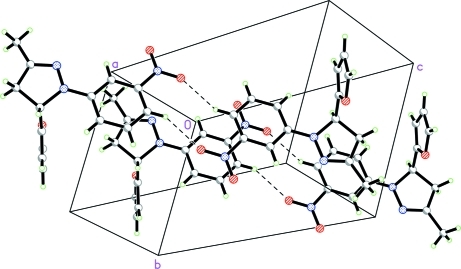
Packing of (I), showing the intermolecular hydrogen bonds as dashed lines.

## supplementary crystallographic information

### Comment

The derivatives of pyrazoline are mostly used in medicine, for example as antitumor (Hatheway *et al.*, 1978), analgesic (Sobczak & Pawlaczyk, 1998), and antimicrobial (Mahajan *et al.*, 1991) agents. As part of our work, the new title compound (I) are synthesized in our group.

The pyrazoline ring assumes an envelope conformation with the furanyl-bearing carbon atom at the flap position (Fig. 1). Intermolecular weak C—H···O hydrogen bonding is present in the crystal structure. (Fig. 2 and Table 1).

### Experimental

3-Nitrophenylhydrazine (1 mmol, 0.153 g) was dissolved in anhydrous ethanol (15 ml). The mixture was stirred for several min at 351 K, furylideneacetone (1 mmol, 0.136 g) in ethanol (8 mm l) was added dropwise and the mixture was stirred at refluxing temperature for 2 h. The product was isolated and recrystallized from methanol, bronze single crystals of (I) were obtained after 3 d.

### Refinement

All H atoms were positioned geometrically and refined as riding with C—H = 0.93 (aromatic), 0.97 (methylene), 0.98 (methine) and 0.96 Å (methyl), with *U*_iso_(H)=1.5*U*_eq_(C) for methyl H atoms and 1.2*U*_eq_(C) for the others.

### Crystal data

**Table:**

C_14_H_13_N_3_O_3_	*Z* = 2
*M**_r_* = 271.27	*F*(000) = 284
Triclinic, *P*1	*D*_x_ = 1.362 Mg m^−^^3^
Hall symbol: -P 1	Mo *K*α radiation, λ = 0.71073 Å
*a* = 6.2089 (2) Å	Cell parameters from 1598 reflections
*b* = 7.8581 (3) Å	θ = 3.5–24.6°
*c* = 14.3800 (4) Å	µ = 0.10 mm^−^^1^
α = 105.764 (2)°	*T* = 296 K
β = 97.054 (2)°	Block, bronze
γ = 96.944 (2)°	0.31 × 0.15 × 0.10 mm
*V* = 661.31 (4) Å^3^	

### Data collection

**Table:**

Bruker SMART CCD area-detector diffractometer	1778 reflections with *I* > 2σ(*I*)
Radiation source: fine-focus sealed tube	*R*_int_ = 0.036
graphite	θ_max_ = 26.0°, θ_min_ = 3.5°
ω scans	*h* = −7→7
9707 measured reflections	*k* = −8→9
2590 independent reflections	*l* = −17→17

### Refinement

**Table:**

Refinement on *F*^2^	Primary atom site location: structure-invariant direct methods
Least-squares matrix: full	Secondary atom site location: difference Fourier map
*R*[*F*^2^ > 2σ(*F*^2^)] = 0.044	Hydrogen site location: inferred from neighbouring sites
*wR*(*F*^2^) = 0.121	H-atom parameters constrained
*S* = 1.10	*w* = 1/[σ^2^(*F*_o_^2^) + (0.0608*P*)^2^] where *P* = (*F*_o_^2^ + 2*F*_c_^2^)/3
2590 reflections	(Δ/σ)_max_ = 0.003
181 parameters	Δρ_max_ = 0.14 e Å^−^^3^
0 restraints	Δρ_min_ = −0.20 e Å^−^^3^

### Special details

**Table:**

Geometry. All e.s.d.'s (except the e.s.d. in the dihedral angle between two l.s. planes) are estimated using the full covariance matrix. The cell e.s.d.'s are taken into account individually in the estimation of e.s.d.'s in distances, angles and torsion angles; correlations between e.s.d.'s in cell parameters are only used when they are defined by crystal symmetry. An approximate (isotropic) treatment of cell e.s.d.'s is used for estimating e.s.d.'s involving l.s. planes.
Refinement. Refinement of *F*^2^ against ALL reflections. The weighted *R*-factor *wR* and goodness of fit *S* are based on *F*^2^, conventional *R*-factors *R* are based on *F*, with *F* set to zero for negative *F*^2^. The threshold expression of *F*^2^ > σ(*F*^2^) is used only for calculating *R*-factors(gt) *etc*. and is not relevant to the choice of reflections for refinement. *R*-factors based on *F*^2^ are statistically about twice as large as those based on *F*, and *R*- factors based on ALL data will be even larger.

### Fractional atomic coordinates and isotropic or equivalent isotropic displacement parameters (Å^2^)

**Table:**

	*x*	*y*	*z*	*U*_iso_*/*U*_eq_	
O	0.36743 (18)	0.34679 (15)	0.90208 (8)	0.0586 (3)	
N1	0.1661 (2)	0.62239 (17)	0.78604 (10)	0.0496 (4)	
N2	0.2000 (2)	0.45740 (16)	0.72794 (10)	0.0491 (4)	
C9	0.3755 (2)	0.4521 (2)	0.67745 (11)	0.0429 (4)	
C14	0.5059 (2)	0.6102 (2)	0.67820 (11)	0.0460 (4)	
H14A	0.4772	0.7211	0.7132	0.055*	
C7	0.0168 (3)	0.5942 (2)	0.83623 (13)	0.0534 (4)	
C10	0.4224 (3)	0.2892 (2)	0.62195 (12)	0.0546 (4)	
H10A	0.3347	0.1826	0.6191	0.066*	
C4	0.2531 (2)	0.2318 (2)	0.81601 (12)	0.0477 (4)	
C13	0.6784 (2)	0.5987 (2)	0.62600 (12)	0.0514 (4)	
N3	0.8105 (2)	0.7665 (2)	0.62761 (12)	0.0680 (5)	
O1	0.9707 (2)	0.7615 (2)	0.58611 (11)	0.0934 (5)	
C5	0.0941 (2)	0.3072 (2)	0.75855 (12)	0.0508 (4)	
H5A	0.0138	0.2126	0.7010	0.061*	
C12	0.7298 (3)	0.4395 (3)	0.57272 (12)	0.0611 (5)	
H12A	0.8487	0.4365	0.5391	0.073*	
C6	−0.0684 (3)	0.4007 (3)	0.81836 (14)	0.0615 (5)	
H6A	−0.2166	0.3686	0.7817	0.074*	
H6B	−0.0677	0.3706	0.8795	0.074*	
C1	0.4967 (3)	0.2476 (3)	0.94299 (14)	0.0607 (5)	
H1B	0.5924	0.2920	1.0022	0.073*	
O2	0.7571 (3)	0.9062 (2)	0.67000 (14)	0.1046 (6)	
C11	0.5976 (3)	0.2848 (3)	0.57136 (13)	0.0642 (5)	
H11A	0.6270	0.1748	0.5355	0.077*	
C3	0.3090 (3)	0.0707 (2)	0.80439 (14)	0.0660 (5)	
H3A	0.2550	−0.0308	0.7518	0.079*	
C2	0.4665 (3)	0.0818 (3)	0.88708 (14)	0.0673 (5)	
H2A	0.5350	−0.0108	0.8992	0.081*	
C8	−0.0654 (3)	0.7433 (3)	0.90245 (15)	0.0793 (6)	
H8A	0.0141	0.8556	0.9019	0.119*	
H8B	−0.0444	0.7329	0.9678	0.119*	
H8C	−0.2191	0.7381	0.8806	0.119*	

### Atomic displacement parameters (Å^2^)

**Table:**

	*U*^11^	*U*^22^	*U*^33^	*U*^12^	*U*^13^	*U*^23^
O	0.0577 (7)	0.0535 (7)	0.0622 (8)	0.0133 (5)	0.0000 (6)	0.0156 (6)
N1	0.0513 (8)	0.0481 (8)	0.0555 (8)	0.0152 (6)	0.0159 (6)	0.0190 (7)
N2	0.0507 (7)	0.0414 (7)	0.0613 (9)	0.0090 (6)	0.0178 (6)	0.0206 (7)
C9	0.0443 (8)	0.0457 (9)	0.0417 (9)	0.0103 (7)	0.0050 (7)	0.0172 (7)
C14	0.0459 (8)	0.0467 (9)	0.0464 (9)	0.0077 (7)	0.0087 (7)	0.0147 (7)
C7	0.0454 (8)	0.0673 (11)	0.0568 (10)	0.0186 (8)	0.0109 (8)	0.0284 (9)
C10	0.0639 (10)	0.0459 (10)	0.0528 (10)	0.0079 (8)	0.0105 (8)	0.0125 (8)
C4	0.0474 (8)	0.0414 (9)	0.0544 (10)	0.0009 (7)	0.0061 (7)	0.0179 (8)
C13	0.0452 (9)	0.0591 (10)	0.0501 (10)	0.0017 (8)	0.0074 (7)	0.0191 (8)
N3	0.0584 (9)	0.0749 (12)	0.0685 (10)	−0.0040 (8)	0.0163 (8)	0.0210 (9)
O1	0.0729 (9)	0.1114 (12)	0.0966 (11)	−0.0083 (8)	0.0418 (8)	0.0290 (9)
C5	0.0440 (8)	0.0488 (9)	0.0621 (10)	−0.0003 (7)	0.0046 (7)	0.0256 (8)
C12	0.0591 (10)	0.0759 (13)	0.0516 (11)	0.0182 (9)	0.0200 (8)	0.0159 (9)
C6	0.0403 (8)	0.0813 (13)	0.0765 (12)	0.0102 (8)	0.0121 (8)	0.0440 (11)
C1	0.0531 (10)	0.0751 (13)	0.0613 (11)	0.0207 (9)	0.0076 (8)	0.0285 (10)
O2	0.0990 (12)	0.0602 (9)	0.1522 (16)	−0.0069 (8)	0.0581 (11)	0.0187 (10)
C11	0.0777 (12)	0.0602 (11)	0.0550 (11)	0.0230 (10)	0.0176 (10)	0.0093 (9)
C3	0.0813 (13)	0.0457 (10)	0.0690 (13)	0.0129 (9)	0.0027 (10)	0.0164 (9)
C2	0.0761 (12)	0.0621 (13)	0.0761 (13)	0.0288 (10)	0.0118 (10)	0.0333 (11)
C8	0.0778 (13)	0.0996 (16)	0.0771 (14)	0.0422 (12)	0.0352 (11)	0.0316 (12)

### Geometric parameters (Å, °)

**Table:**

O—C4	1.3689 (18)	N3—O2	1.208 (2)
O—C1	1.372 (2)	N3—O1	1.2209 (19)
N1—C7	1.277 (2)	C5—C6	1.532 (2)
N1—N2	1.3940 (18)	C5—H5A	0.9800
N2—C9	1.3803 (19)	C12—C11	1.376 (3)
N2—C5	1.4784 (19)	C12—H12A	0.9300
C9—C14	1.396 (2)	C6—H6A	0.9700
C9—C10	1.397 (2)	C6—H6B	0.9700
C14—C13	1.379 (2)	C1—C2	1.309 (2)
C14—H14A	0.9300	C1—H1B	0.9300
C7—C8	1.482 (2)	C11—H11A	0.9300
C7—C6	1.489 (3)	C3—C2	1.420 (2)
C10—C11	1.380 (2)	C3—H3A	0.9300
C10—H10A	0.9300	C2—H2A	0.9300
C4—C3	1.325 (2)	C8—H8A	0.9600
C4—C5	1.488 (2)	C8—H8B	0.9600
C13—C12	1.375 (2)	C8—H8C	0.9600
C13—N3	1.459 (2)		
			
C4—O—C1	105.97 (13)	C4—C5—H5A	110.0
C7—N1—N2	108.34 (13)	C6—C5—H5A	110.0
C9—N2—N1	118.80 (12)	C13—C12—C11	117.03 (16)
C9—N2—C5	125.31 (13)	C13—C12—H12A	121.5
N1—N2—C5	111.64 (12)	C11—C12—H12A	121.5
N2—C9—C14	120.55 (14)	C7—C6—C5	103.00 (13)
N2—C9—C10	120.96 (14)	C7—C6—H6A	111.2
C14—C9—C10	118.47 (14)	C5—C6—H6A	111.2
C13—C14—C9	118.59 (15)	C7—C6—H6B	111.2
C13—C14—H14A	120.7	C5—C6—H6B	111.2
C9—C14—H14A	120.7	H6A—C6—H6B	109.1
N1—C7—C8	121.87 (16)	C2—C1—O	110.60 (15)
N1—C7—C6	113.46 (15)	C2—C1—H1B	124.7
C8—C7—C6	124.64 (16)	O—C1—H1B	124.7
C11—C10—C9	120.67 (16)	C12—C11—C10	121.50 (17)
C11—C10—H10A	119.7	C12—C11—H11A	119.3
C9—C10—H10A	119.7	C10—C11—H11A	119.3
C3—C4—O	109.50 (15)	C4—C3—C2	107.27 (16)
C3—C4—C5	134.10 (17)	C4—C3—H3A	126.4
O—C4—C5	116.37 (14)	C2—C3—H3A	126.4
C12—C13—C14	123.72 (16)	C1—C2—C3	106.65 (16)
C12—C13—N3	119.04 (15)	C1—C2—H2A	126.7
C14—C13—N3	117.24 (15)	C3—C2—H2A	126.7
O2—N3—O1	122.13 (17)	C7—C8—H8A	109.5
O2—N3—C13	118.76 (15)	C7—C8—H8B	109.5
O1—N3—C13	119.11 (17)	H8A—C8—H8B	109.5
N2—C5—C4	112.93 (12)	C7—C8—H8C	109.5
N2—C5—C6	100.18 (13)	H8A—C8—H8C	109.5
C4—C5—C6	113.50 (14)	H8B—C8—H8C	109.5
N2—C5—H5A	110.0		

### Hydrogen-bond geometry (Å, °)

**Table:**

*D*—H···*A*	*D*—H	H···*A*	*D*···*A*	*D*—H···*A*
C12—H12A···O1^i^	0.93	2.51	3.311 (2)	144

Symmetry codes: (i) −*x*+2, −*y*+1, −*z*+1.
